# Global left ventricular remodeling, extent of inferior wall infarct, and mitral valve geometry are important predictors of mitral regurgitation severity than total infarct size in advanced ischemic cardiomyopathy

**DOI:** 10.1186/1532-429X-16-S1-P229

**Published:** 2014-01-16

**Authors:** Joao L Cavalcante, Nancy Obuchowski, Qusai Saleh, Zoran Popovic, Milind Y Desai, Scott Flamm, Deborah Kwon

**Affiliations:** 1 Cardiology, University of Pittsburgh, Pittsburgh, Pennsylvania, USA; 2 Radiology - Cardiovascular Imaging, Cleveland Clinic, Cleveland, Ohio, USA; 3 Internal Medicine, Cleveland Clinic, Cleveland, Ohio, USA; 4 Heart & Vascular Institute, Cleveland Clinic, Cleveland, Ohio, USA

## Background

Mitral regurgitation (MR) is common in patients with ischemic cardiomyopathy (ICM) and independently associated with worse mortality. We sought to determine the impact of adverse LV remodeling, total myocardial infarct (MI) size, location and extent of regional MI, and mitral valve geometry on the severity of mitral regurgitation.

## Methods

A total of 494 patients with LVEF ≤ 40% and ≥ 70% stenosis in ≥ 1 epicardial coronary artery and prior history of CABG or PCI were screened. Patients with transthoracic echocardiogram and CMR studies within 7 days were selected. Forty-two patients (9%) were excluded either due to prior mitral valve surgery, unavailable studies or organic mitral valve disease. Other 42 patients were excluded because of no mitral regurgitation noted on their study. Mitral regurgitation was assessed using semi-quantitative vena contracta method from a parasternal long axis zoomed view obtained of the mitral valve. LV volumes and EF were calculated from cine short axis CMR images. Mitral valve geometry was measured on end-systolic 2, 3, and 4 chamber cine views. LV scar was measured 10-15 minutes after gadolinium injection and using phase-sensitive inversion recovery sequence.

## Results

**Figure 1:**
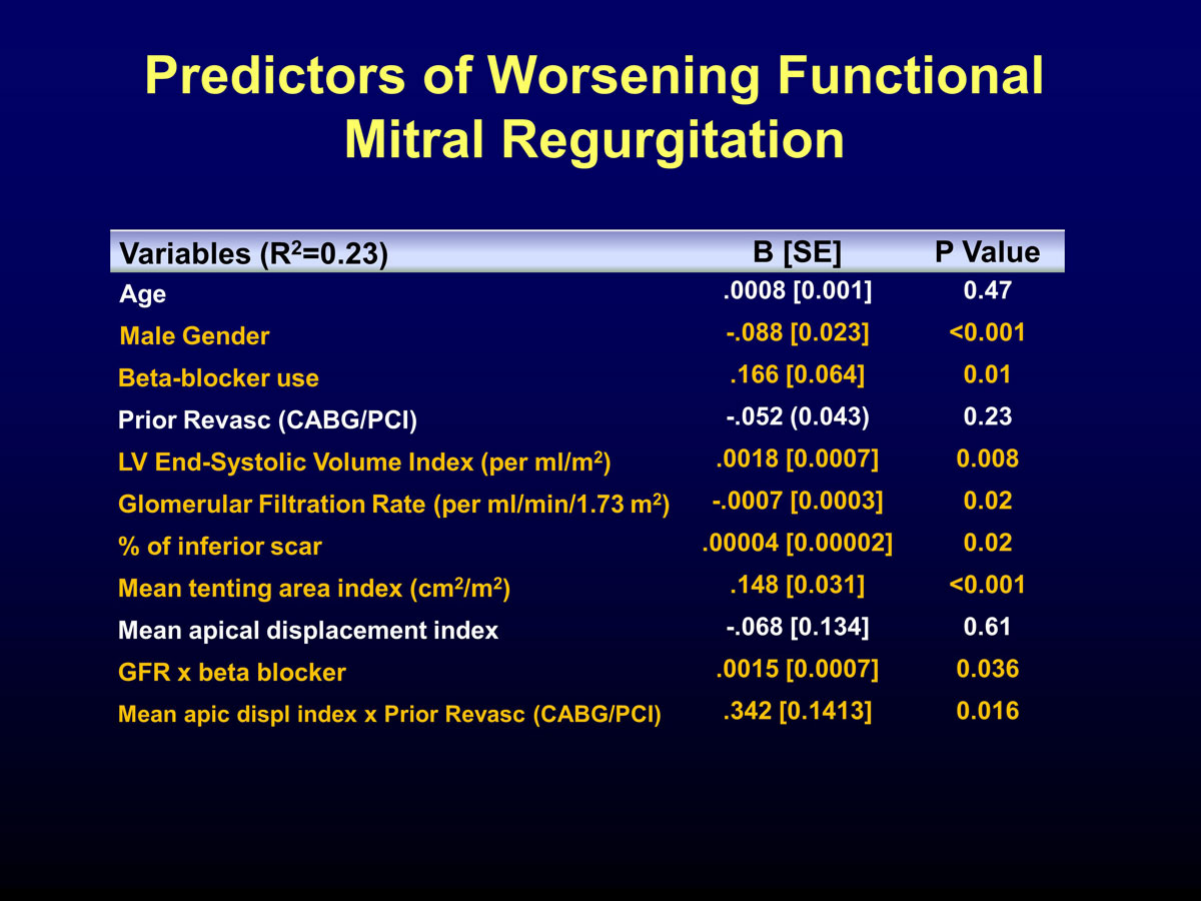
**Predictors of worsening functional mitral regurgitation**.

The mean age of the cohort (n = 410) was 63 ± 11 years and males represented 75%. LVEF, LV end-systolic volume index, mean total infarct size, MV annulus index, MV tenting area index, and MV displacement index were: 23 ± 0.5%, 115 ± 2 ml/m2, 31 ± 17%, 1.7 ± 0.5 cm2/m2, 0.88 ± 0.48 cm2/m2, respectively. Mean inferior, lateral, anterior infarct size were: 27 ± 21%, 25 ± 20%, 37 ± 23%. MR was classified as: mild (63%), moderate (22%) or severe (15%). The mean vena contracta was 0.33 ± 0.24 cm. Multiple variable linear regression (R2 = 0.23) revealed that male gender, worsening GFR, increase extent of inferior LV scar, LV ESVi and MV tenting area index were independent predictors of MR severity, whereas overall scar extent, anterior or lateral location were not. (Figure 1)

## Conclusions

Global LV remodeling, regional inferior myocardial scarring, and mitral valve geometry are more important predictors of worsening MR than total infarct size in patients with advanced ischemic cardiomyopathy.

## Funding

None.

